# Exsanguination of a home hemodialysis patient as a result of misconnected blood-lines during the wash back procedure: A case report

**DOI:** 10.1186/1471-2369-13-28

**Published:** 2012-05-15

**Authors:** Kerryanne Allcock, Balaji Jagannathan, Christopher J Hood, Mark R Marshall

**Affiliations:** 1 Department of Renal Medicine, Middlemore Hospital, Counties Manukau District Health Board, Private Bag 93311, Otahuhu, Auckland, 1640, New Zealand; 2 Renal Service, North Shore Hospital, Waitemata District Health Board, Private Bag 93503, Takapuna, Auckland, 0740, New Zealand

**Keywords:** Home hemodialysis, Exsanguination, Complication

## Abstract

**Background:**

Home hemodialysis is common in New Zealand and associated with lower cost, improved survival and better patient experience. We present the case of a fully trained home hemodialysis patient who exsanguinated at home as a result of an incorrect wash back procedure.

**Case presentation:**

The case involves a 67 year old male with a history of well controlled hypertension and impaired glucose tolerance. He commenced on peritoneal dialysis in 2006 following the development of end stage kidney failure secondary to focal segmental glomerulosclerosis. He transferred to hemodialysis due to peritoneal membrane failure in 2010, and successfully trained for home hemodialysis over a 20 week period. Following one month of uncomplicated dialysis at home, he was found deceased on his machine at home in the midst of dialysis. His death occurred during the wash back procedure performed using the “open circuit” method, and resulted from misconnection of the saline bag to the venous end of the extracorporeal blood circuit instead of the arterial end. This led to approximately 2.3L of his blood being pumped into the saline bag resulting in hypovolaemic shock and death from exsanguination.

**Conclusions:**

Despite successful training, critical procedural errors can still be made by patients on home hemodialysis. In this case, the error involved misconnection of the saline bag for wash back. This case should prompt providers of home hemodialysis to review their training protocols and manuals. Manufacturers of dialysis machinery should be encouraged to design machines specifically for home hemodialysis, and consider distinguishing the arterial and venous ends of the extracorporeal blood circuit with colour coding or incompatible connectivity, to prevent occurrences such as these in the future.

## Background

For those with end stage kidney failure, hemodialysis is the most common modality of treatment. Haemodialysis is commonly performed at home in Australasia and particularly in New Zealand. This practice is motivated by better clinical and patient experience, improved patient outcomes, and lower cost [1-6]. Our nephrology service is based in Auckland, New Zealand, and has been providing home hemodialysis for almost 30 years. Currently, we care for approximately 80 prevalent home hemodialysis patients and train between 20 and 25 patients for home hemodialysis per year. Such patients are selected for home hemodialysis by a multidisciplinary team. While there are no absolute inclusion or exclusion criteria for home hemodialysis, there must be general agreement within the multidisciplinary team that patients are medically, physically, and functionally capable. Once they are selected, training for home hemodialysis is rigorous and lasts on average 110 days. After that, patients perform their hemodialysis at home with frequent phone support and monthly home visits from their trainer/case manager.

**Figure 1:**
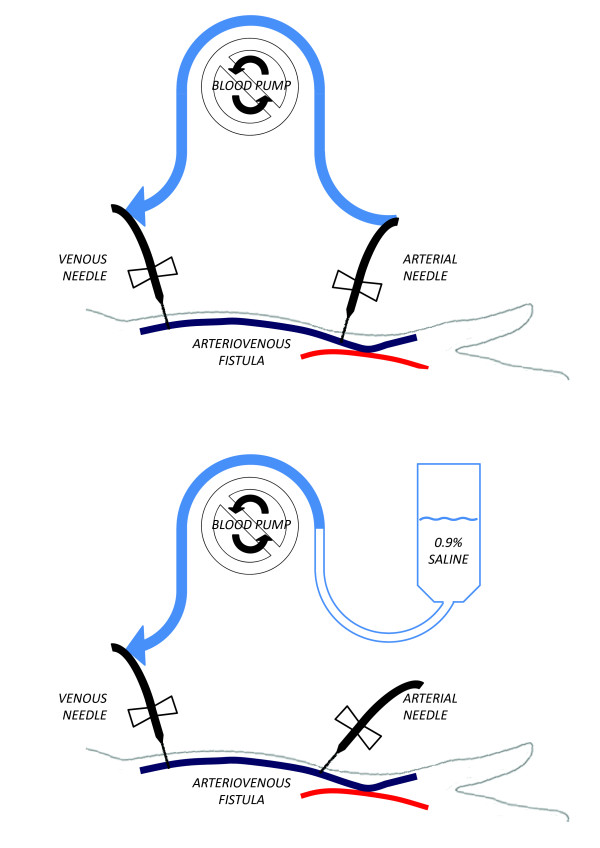
**Schematic of normal extracorporeal blood circuit configuration.** The top panel represents configuration during a normal hemodialysis treatment. The bottom panel represents configuration during the wash back procedure using the “open circuit” method with the arterial end of the circuit connected to the saline infusion set.

An important aspect of hemodialysis is the wash back procedure at the end of treatment. During this procedure, saline is infused through the dialysis machine’s extracorporeal blood circuit thereby returning blood to the patient’s circulation and minimizing blood loss. In our home hemodialysis training program, we instruct patients to use the “open circuit” method. The correct form of this procedure is illustrated in Figure 1. Summarily, the procedure involves disconnecting the arterial end of the extracorporeal blood circuit from the arterial needle, then connecting the arterial end of the circuit to a saline infusion set, before using the blood pump to draw saline through the extracorporeal blood for wash back.

We present a case of a patient who died performing his hemodialysis at home as a result of an incorrect wash back procedure.

## Case presentation

The case involves a male who was 67 years old at the time of his death. He was a retired businessman, married with several children, and socioeconomically advantaged. He lived with his wife and children, and his mother. He spoke both English and Hindi fluently, and had no sensory or cognitive impairment. His only co-morbidities were longstanding well controlled hypertension and impaired glucose tolerance.

He was diagnosed with chronic kidney disease secondary to focal segmental glomerulosclerosis in 2003. He commenced peritoneal dialysis for end stage kidney failure in 2006, which continued without any infectious or technical complication for the entire period of his treatment with this modality. He developed peritoneal membrane failure after 4 years; we made a decision to transition him to home hemodialysis in early 2010.

He had an arteriovenous fistula placed on 21^st^ July 2010, and commenced home hemodialysis training 14^th^ September 2010. He completed training on 7^th^ February 2011, and commenced home hemodialysis thereafter. There were no reported problems or technical difficulties throughout his training. His hemodialysis prescription was for 5 hours of dialysis three times a week, using a hollow fibre polyamide 2.1m^2^ high flux dialyser, bicarbonate-based dialysate with a [K+] of 1.25 mM, and a Gambro AK200S machine (Gambro AB, Lund, Sweden).

On the day of this death, 3^rd^ March 2011, the patient had contacted his case manager in the morning to propose a decrease in his target weight and the removal of more fluid than prescribed. He was advised against this. Several hours later, his family called the unit to report that they had returned home to discover him dead while still attached to his dialysis machine.

**Figure 2:**
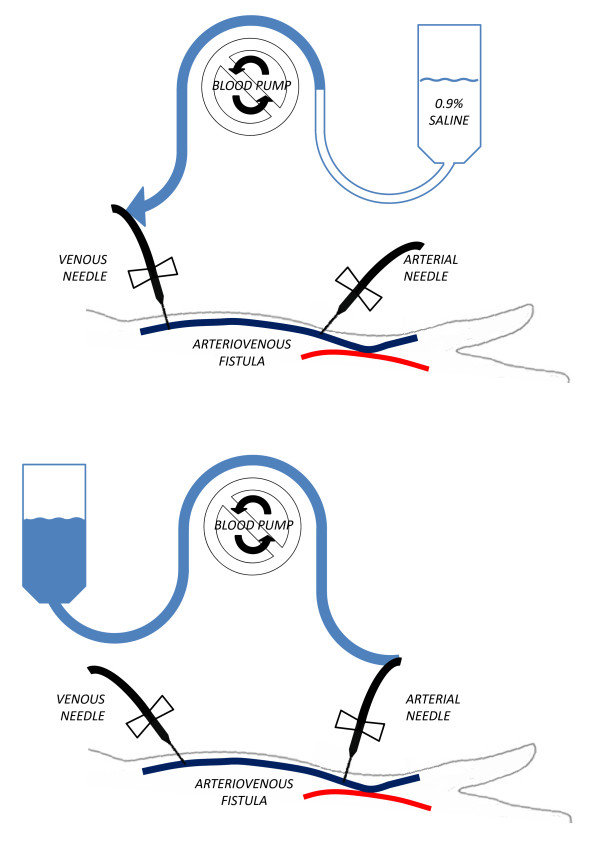
**Schematic of extracorporeal blood circuit configuration in this case.** The top panel represents configuration during a normal wash back procedure using the “open circuit” method. The lower panel represents the configuration of the patient’s circuit with the venous end of the circuit connected to the saline infusion set.

**Figure 3:**
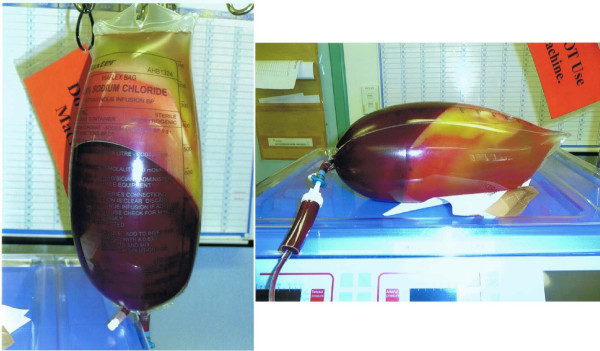
**The saline bag found attached to the deceased’s dialysis machine.** The weight of the bag was 3.3kg, implying the addition of 2.3L of the patient’s blood to the bag of saline during the wash back procedure.

Investigations showed that the patient had exsanguinated during the wash back procedure. During the wash back, a 1 L bag of saline had been misconnected to the venous end of the extracorporeal blood circuit, rather than the arterial end. As a result, the patient’s blood was pumped out of his circulation into the bag of saline, instead of saline being pumped out of the bag through the extracorporeal blood circuit (Figure 2). The bag of saline is shown in Figure 3, and weighed 3.3 kg implying at least 2.3 L of the patient’s blood in addition to 1 L of saline. Of note, his last blood pressure reading was recorded one and half hours before the end of dialysis (patients are usually advised to take their observations hourly), and at that time his blood pressure and heart rate were satisfactory (146/84 and 59 bpm respectively).

**Figure 4:**
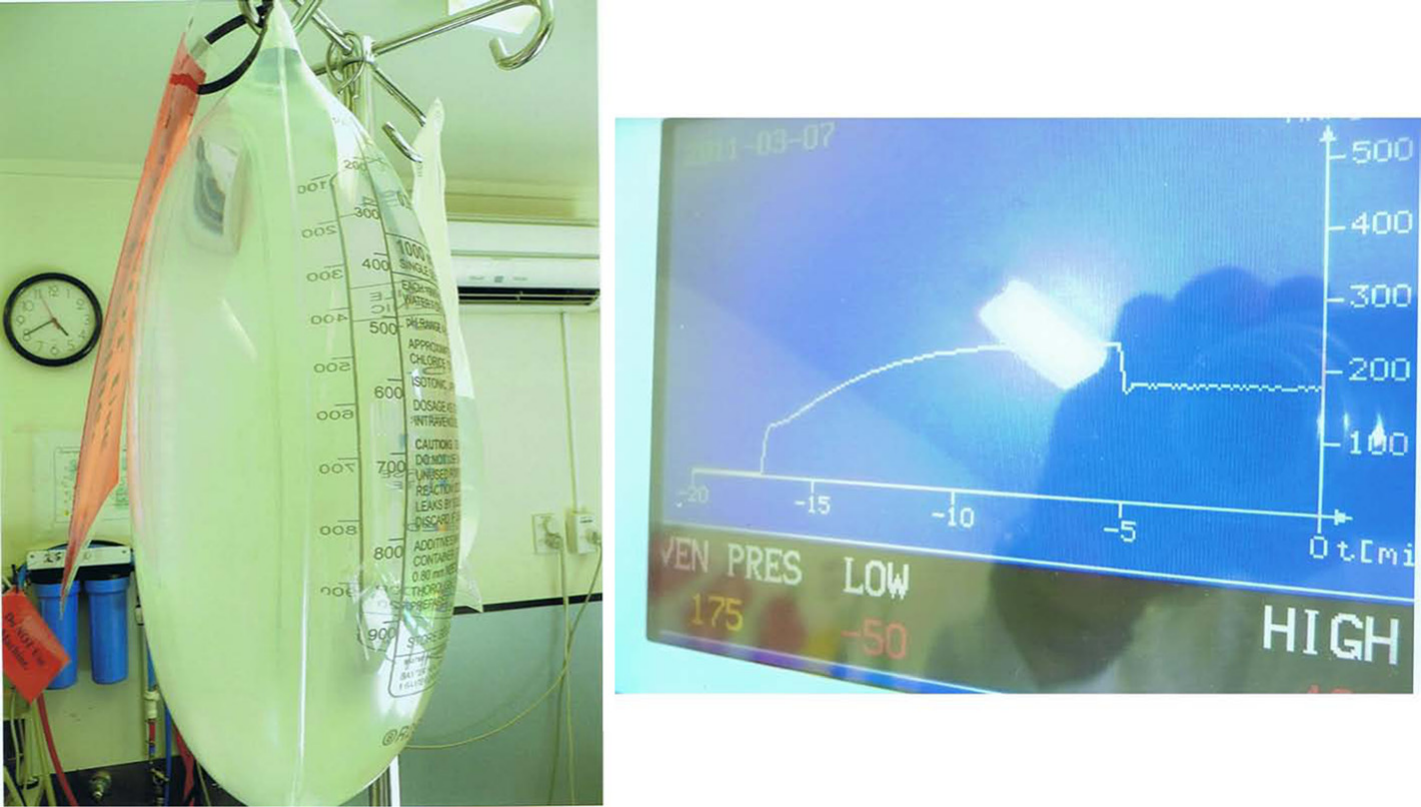
**A saline bag after a simulation of circumstances surrounding the deceased’s last dialysis treatment.** The weight of the bag was 4kg, and venous pressures with a pump speed of 200mL/min rose to only just higher than 200mmHg during this exercise.

Exhaustive testing of the patient’s hemodialysis machine showed that it was functioning correctly, and in particular venous alarms were triggering normally. To replicate the patient’s situation, we tested the distensibility of a bag of normal saline and determined that it can expand to several times its volume to accommodate at least three litres of extra fluid, all without substantial increase in venous pressure on the dialysis machine or bag rupture (Figure 4).

At a blood pump speed of 200 mL/min, it would have taken more than 10 minutes to fill the saline bag with as much blood as we found. Consequently, we were concerned that the patient may have been acutely incapacitated by a primary cardiovascular or cerebrovascular event at the time of the wash-back. A post mortem examination was performed, with the forensic pathologist reporting no specific anatomic, toxicological or biochemical abnormality to account for his death. Minor arthrosclerosis was noted in the left anterior descending and right coronary arteries, left vertebral artery, and the infrarenal aorta. The myocardium and brain were normal on multiple sections. The Coroner’s conclusion was that death was due to hypovolaemic shock arising from blood loss into an incorrectly connected saline bag.

Three providers of home hemodialysis around New Zealand have reported similar incidents. Two occurred in setting of home hemodialysis training rather than dialysis at home. In both of these cases, the trainer noticed the error immediately and was able to rectify the problem and use it as a teaching point. The third case occurred under ostensibly similar circumstances to ours, also appearing to lead to the patient’s death. That case is currently sub judice with the Coroner’s Office.

The death of our patient prompted an external formal review of our home hemodialysis training program, resulting in the following changes. We now make patients aware of this specific potential complication, and emphasize to them that they must ensure clearing of the extracorporeal blood circuit after a minute or two of the wash back procedure. We also emphasize the potential dangers of performing home hemodialysis alone, especially in those who are newly trained, and re-iterate our recommendation that there is someone else in the home when they dialyse if that is possible. Finally, we have developed a one-way valve that we are incorporating into the saline line for all of our home hemodialysis patients. This valve prevents reflux of blood back into the saline bag from the extracorporeal blood circuit in the event of misconnection, and triggers pressure alarms that alert patients to their error.

**Figure 5:**
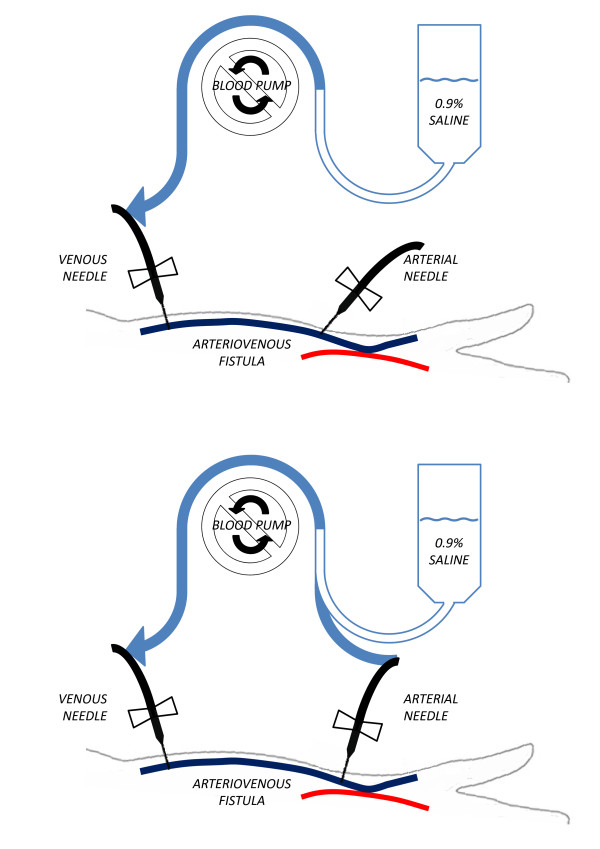
**Schematic of wash back procedures at the discontinuation of dialysis.** The “open circuit” method is illustrated in the top panel in the top panel, and the “closed circuit” method in the bottom panel.

Another potential solution is the “closed circuit” wash back procedure. In this procedure, there is a Y-connection close to the arterial end of the extracorporeal blood circuit where it attaches to the arterial needle. At the discontinuation of dialysis, the patient attaches the saline line (or on-line ultrapure/sterile dialysate line) to the Y-connect, then clamps the line between the arterial needle and the Y-connect, before using the blood pump to draw saline through the extracorporeal blood circuit for wash back. The procedure is illustrated in Figure 5. The primary shortcoming of this procedure pertains to the residual blood between the arterial needle and the Y-connect, usually 2.5-5mL depending on manufacturer. This blood can either be discarded, or returned to the patient by stopping the blood pump and allowing gravity to motivate saline from the hanging bag towards the arterial needle in a retrograde fashion through the circuit. This effectiveness of the last part of this procedure is limited by the opposing pressure in arteriovenous access, and in our experience is not successful in many patients without pressure applied to the saline bag which is generally not a standard or recommended practice.

The manufacturer of our patient’s dialysis machine is aware of this incident. There have been discussions around the feasibility of changing the extracorporeal blood circuit to make such an error impossible in the future. For instance, the arterial and venous ends of the circuit could be produced with different coloured or incompatible connections to make it difficult for patients to misconnect them. Currently, this fail-safe facility is not available from any manufacturer of dialysis machines.

## Conclusion

Home hemodialysis is a durable and effective form of renal replacement therapy. Despite this, there are a myriad of complications that can occur [7]. The particular complication that we have described is the first report in the medical literature as far as we are aware. Providers of home hemodialysis services and hemodialysis trainers should emphasize this potential complication to patients and design wash-back procedures that will detect or prevent misconnections. We feel strongly that manufacturers should be designing hemodialysis machines that are specifically suited for home hemodialysis. In our opinion, such a machine would include arterial and venous ends of the extracorporeal blood circuit with colour coded or incompatible connections to prevent future incidents such as these.

## Consent

Written consent was obtained from the patient’s family for the publication of the case report and its accompanying images.

## Competing interests

The authors declared that they have no competing interests.

## Authors’ contributions

MM and CH treated the patient, MM and BJ collected data and drafted the manuscript together with KA. All authors read and approved the final manuscript.

## Authors’ information

KA (MBBS) is a Renal Registrar at Counties Manukau District Health Board; BJ (BSc PGDip Dialysis Therapy), is the Head Clinical Dialysis Technician at Waitemata District Health Board; CJH (MA MBBS FRACP) is a nephrologist at Counties Manukau District Health Board; MRM (MPH (Hons) FRACP) is a nephrologist and the Clinical Director of Renal Medicine at Counties Manukau District Health Board.

## Pre-publication history

The pre-publication history for this paper can be accessed here:

http://www.biomedcentral.com/1471-2369/13/28/prepub
